# Socioeconomic disparities in using rehabilitation services among Iranian adults with disabilities: a decomposition analysis

**DOI:** 10.1186/s12913-022-08811-8

**Published:** 2022-11-29

**Authors:** Shahin Soltani, Marzieh Mohammadi Moghadam, Shiva Amani, Shahram Akbari, Amir Shiani, Moslem Soofi

**Affiliations:** 1 Research Center for Environmental Determinants of Health (RCEDH), Health Institute, Kermanshah University of Medical Sciences, Kermanshah, Iran; 2 Sabzevar University of Medical Sciences, Sabzevar, Iran; 3 Students Research Committee, Kermanshah University of Medical Sciences, Kermanshah, Iran; 4 Clinical Research Development Center, Taleghani and Imam Ali Hospital, Kermanshah University of Medical Sciences, Kermanshah, Iran; 5 Social Development and Health Promotion Research Center, Health Institute, Kermanshah University of Medical Sciences, Kermanshah, Iran

**Keywords:** Socioeconomic factors, Inequality, Concentration index, Rehabilitation, Disability, Iran

## Abstract

**Background:**

Persons with disabilities (PWD) generally experience various barriers in using health care compared to the general population, and these problems are more worsened for those with disabilities in lower socioeconomic status. The study aimed to estimate socioeconomic inequality in using rehabilitation services (URS) in adults with disabilities in Iran.

**Methods:**

This cross-sectional study was conducted at a national level in Iran. 786 PWD (aged 18 years and older) participated in the study between September and December 2020. Socioeconomic-related inequality in URS was estimated by the Concentration Index (C). The C was decomposed to identify factors explaining the variability within the socioeconomic inequality in URS.

**Results:**

In the present study 8.10% (*N* = 61) of the study population used rehabilitation services during the past three months. In this study, the value of the C was estimated 0.25 (*p*-value = 0.025) that shows URS was unequally distributed, and concentrated among the higher SES groups. The results of decomposition analysis indicated that the wealth index was the largest contributor (94.22%) to the observed socioeconomic inequalities in URS among PWD. Following the wealth index, Age and marital status were the major contributors to the unequal distribution of URS among the study population.

**Conclusions:**

Our findings revealed that socioeconomic inequality in using rehabilitation services was concentrated among well-off PWD. Accordingly, rehabilitation financing through appropriate mechanisms for individuals with low SES is suggested.

## Introduction

Universal health coverage is about ensuring that all members of the population and their communities have access to promotive, preventive, curative, rehabilitative and palliative health services without suffering financial hardship. However, persons with disabilities (PWD) generally experience major obstacles to access to healthcare compared with the general population. PWD face a wide range of physical, geographical, cultural and financial barriers to health care access [1–3]. Of these, PWD in lower socioeconomic groups are more likely to report poorer access to health services than those with higher socioeconomic status (SES) [4]. These barriers can result in adverse health outcomes and health disparities between PWD and the general population [5].

Disability is an important public health issue in Iran where about 1–4% of Iranian population live with a disability [6, 7]. In contrast, some studies report different amounts for the prevalence of disability compared to the previous national censuses in Iran. For example, in a population based study in Tehran, the prevalence of disability among Iranian older adults over 60 years old was 11% in which hearing loss was the most common type of disability (85%). In another study on 470 elderly people aged 60 and over, in Gonabad city in Iran, 40.6% of the elderly experienced low disability, 15.2% had moderate disability and 8.6% face severe disability  [8].

In the Comprehensive Act on the Protection of the Rights of People with Disabilities (CAPRPD) in Iran 2002, disability is defined as continuous and significant physical, mental, psychological or combined disorders in health and general functioning which reduce the independence in activities of daily living. In recent decades there have been significant advances in providing rehabilitation and social services (disability pension, free rehabilitation services, special vehicles, and assistive devices, etc.) for PWD in Iran, but they still face different obstacles to use healthcare [9–11].

Previous studies in Iran indicate that sociocultural factors (e.g. negative attitudes, reluctance to provide health care for PWD, disrespect, discrimination, lack of awareness, misconceptions) [3, 12] physical barriers [13, 14], geographical features, and financial problems are major contributors to the poor access to medical services in Iran [2].

In this article, we focus on socioeconomic inequalities in using rehabilitation services (URS) among adults with disabilities in Iran. World Health Organization defines rehabilitation as *“*a set of interventions designed to optimize functioning and reduce disability in individuals with health conditions in interaction with their environment “ [15]. Given the kind and the severity of disabilities, PWD may be need different emergency medical and rehabilitation services [16].

In Iran, rehabilitation services (e.g. physical medicine, physical therapy, audiology, speech therapy, occupational therapy, orthotics and prosthetics) are provided in various settings such as inpatient setting (1.683 rehabilitation beds per 1,000,000 population), outpatient setting (89.243 per 1,000,000 population) and community-based settings (66.214 for every million people), and long-term care setting ( 3.603 nursing home centers per 1,000,000 population) [17]. Also, rehabilitation services are provided in different sections such as public, private, and non-governmental centers. According to a study by Shirazikhah et al. (2017), in Iran, physiotherapists (64.650 per 1,000,000 population), audiologists (24.185 per 1,000,000 population), speech therapists (22.830 per 1,000,000 population), occupational therapists (22.094 per 1,000,000 population), and physical medicine and rehabilitation specialist (3.905 per 1,000,000 population) consist of the main members of a rehabilitation team in Iran [17].

Some services such as occupational therapy, speech therapy, and orthotics and prosthetics are not covered by health insurance that imposes high out of pocket payments (OOP) on PWD in Iran. In addition, physiotherapy, as an essential service to improve functioning is associated with co-pay that families in lower SES groups may not afford to pay for OOP. A study by Rezaei et al. in Iran reveals that the OOP for healthcare is regressive and more concentrated in lower SES groups [18]. The findings of Zarei et al. demonstrate that the OOP share for physiotherapy is around 31% in Iran [19]. Similarly, Ayobian et al. noted that the high cost of physical therapy can cause less use of this service in people with spinal cord injuries in Iran [20]. Despite previous studies, evidence is lacking about socioeconomic inequalities in URS using inequality indicators for PWD. Previous studies have used qualitative analyses or quantitative methods such as parametric tests and regression models to identify determinants of access to healthcare in Iran [21–25]. In this study, we applied concentration index (C) to estimate socioeconomic related health inequalities in URS for adults with disabilities in Iran. The C is the most suggested method to analyze binary health variables encountered in healthcare research and many health economics.

## Method

### The study population

The samples in this observational cross-sectional study were recruited from the Iranian Society with Disabilities (ISD). The ISD is a non-governmental organization (NGO) that facilitates access to education and healthcare services for PWD in all provinces of Iran [26]. At the time of the present study, around 50,000 adults with disabilities (age >  = 18 years old) were members of the ISD. This NGO has 19 branches and 47 local offices in all over Iran, and provides various social, financial, educational, and healthcare supports for adults with disabilities particularly those in lower socioeconomic status. We used convenience sampling to include participants in the study.

The inclusion criteria in the present study consisted of:

1. PWD aged 18 years and older
2. People who were the member of ISD
3. People with Iranian nationality

Additionally, the exclusion criteria included:

1. People who were reluctant to participate in the study
2. People aged under 18 years
3. People without disabilities

### Data collection

To measure socioeconomic inequalities in URS, we used a valid and reliable questionnaire developed by Karami Matin et al. in a previous study [27]. This short and self-report questionnaire collects demographic information, SES, and access to rehabilitation services. Also, we used the Washington Group general measure to determine disability status [28]. Questions have been developed according to the most basic level of functioning: hearing, seeing, walking or climbing steps; remembering or concentrating; washing all over or dressing, and communicating. The six functions were adopted as universal, happening generally and related to social exclusion. Also, the severity of functional limitation in each item was assessed by a four-point scale: “no difficulty”, “some difficulty”, “a lot of difficulty”, and “unable to do it”. Further details about this questionnaire have been reported by Palmer and Harley [28].

Given the pandemic of COVID-19, we decided to apply an electronic form to collect data. Thus, we sent a shared link of the electronic questionnaire via messenger applications (Telegram and WhatsApp) to the members of the ISD. We gathered data between September and December 2020.

### Variables

The outcome variable was a binary variable showing whether the participant used rehabilitation services during the past three months or not. Also, demographic variables (age, gender, place of residence, marital status, head of household status, health insurance coverage), disability severity, and socioeconomic factors were included in our analysis as determinants of URS.

Although income apparently is the main indicator of wealth that can be applied as a measure of economic status, it is very difficult to capture exactly [29]. In the 1990s, Filmer and Pritchett found that household features and material assets were much clearer to obtain and could be utilized as a proxy for consumption and, as a result, for economic status [30]. This resulted in the development of the wealth index [31]. Different studies have used the wealth index to investigate subjects such as obesity [32, 33], physical activity [34, 35], oral health [36], malaria transmission [37], reproductive health [38], and poverty [39].

With regard to existing data, we used information on assets ownership (e.g., owning car, stove, refrigerator, vacuum machine, personal computer, and washing machine), housing characteristics (e.g. private or rental house, house area) and educational level of participants to develop the SES variable. Therefore, the SES indicator was created by a combination of households' assets and education levels of participants. To develop the SES indicator, we used principal components analysis (PCA) [40–42]. This technique was used to reduce multi-dimensional data sets on ownership of various household assets to a lower number of dimensions. Thus, the study population were divided into five SES quintile from the lowest (1st quintile) to the highest (5th quintile) SES groups.

### Statistical analysis

## Socioeconomic-related inequality in using rehabilitation services

In the present study, the concentration index (C) was used to measure socioeconomic related inequality in URS among the study population [43]. The C estimates inequality in one variable over the distribution of another. This index is a particularly popular option for the measurement of socioeconomic-related health inequality so that there are more than 9,220 records in Google Scholar with the keywords ‘concentration index’ and ‘health’ [29, 44, 45]. The C is according to the concentration curve that plots the cumulative percentage of the outcome variable (URS) on the vertical axis and the cumulative percentage of the population ranked by their SES on the horizontal axis beginning with the lowest and ending with the highest. The C is twice the area between the concentration curve and the line of equality (the 45-degree line). The amount of C varies between -1 and + 1. The positive value of the C indicates the concentration of the health outcome among high SES groups and vice versa. The zero amount of the C indicates the equal socioeconomic distribution of the health outcome among the different SES groups. The C is calculated by the following “convenient covariance” formula [46]:

$$C=\frac{2*cov({y}_{i} {r}_{i}) }{\mu }, (1)$$

where ${y}_{i}$ is the health outcome variable (i.e., URS) for participant$i$, ${r}_{i}$ is the fractional rank of participant $i$ in the distribution of SES indicator, $\mu$ is the mean of the health outcome variable. Since URS was a binary variable, the maximum and minimum of the C are not + 1 and -1. Thus, the C was normalized according to the Wagstaff’s method [47]:

2 $${C}_{n}=\frac{1}{1-\mu }$$

## Decomposition of socioeconomic inequality in using rehabilitation services

The estimated value of the normalized C was decomposed to identify the contribution of explanatory variables to the observed socioeconomic inequality in URS [48]. Wagstaff and colleagues [48] indicated that if we have a regression model relating a health outcome variable of $y$ to a set of $k$ explanatory variables, $x$ such as:

$$y=\alpha +\sum_{k}{\beta }_{k} {x}_{k}+ \varepsilon , (3)$$

the C for $y$ can be decomposed as:

$$C=\sum_{k}\left( \frac{{\beta }_{k}{\overline{x} }_{k}}{\mu }\right){C}_{k}+G{C}_{\varepsilon }/\mu . (4)$$

In this equation, ${\overline{x} }_{k}$ indicates the mean of the explanatory variable,$x$, ${C}_{k}$ is the C for each explanatory variable, and $G{C}_{\varepsilon }$ shows the generalized C for $\varepsilon$. In Eq. 4, the first component $\sum_{k}\left( \frac{{\beta }_{k}{\overline{x} }_{k}}{\mu }\right){C}_{k}$ indicates the contribution of explanatory variable $x$ to the overall socioeconomic-related inequality in the outcome variable. The contribution of each explanatory variable is a product of two components: (i) its impact on URS, as measured by $\left( \frac{{\beta }_{k}{\overline{x} }_{k}}{\mu }\right)$, and (ii) its degree of unequal distribution across SES groups, as measured by the (C_k_). If the value of the contribution of variable X is x and positive (negative), then, if the variable had no impact on URS or were equally distributed across the SES groups, inequality in URS would decrease (increase) by x%. Also, in Eq. 4, the second component, $\frac{G{C}_{\varepsilon }}{\mu }$ indicates the proportion of socioeconomic inequality in URS which is not explained by the systematic variation of the included explanatory variables across SES groups. Applying Wagstaff’s correction into Equation [47] results in:

5 $${C}_{n}=\frac{C}{1-\mu }=\frac{\sum_{k}\left(\frac{{\beta }_{k}{\overline{x} }_{k}}{\mu }\right){C}_{k}}{1-\mu }+\frac{G{C}_{\varepsilon }/\mu }{1-\mu }$$

Regarding that URS was a binary variable, we used marginal effects derived from a logistic model as $\beta$ in the decomposition of the C. In this study, data was analyzed using Stata version 14.2 (StataCorp, College Station, TX, USA).

## Results

Regarding Table 1, 786 PWD aged 18 to 70 years participated in the present study. The mean age of the study participants was 36.47 years ± 10.02. In this study, 64.36% (*N* = 493), 40.34% (*N* = 309), and 88.33% (*N* = 681) of participants were male, married and lived in the urban setting, respectively. Also, 43.88% (*N* = 344) of participants were covered by Social Security Insurance, and 55.75% (*N* = 441) had an academic degree. In the present study, 8.10%( *N* = 61) of the study population used rehabilitation services during the last month. Also, 46.22%( *N* = 348) of participants had difficulty seeing, 17.2%( *N* = 123) hearing, 73.05%( *N* = 561) walking, 39.36%( *N* = 447) self-caring, 29.9%( *N* = 211) remembering or concentrating, and 21.19%( *N* = 157) communicating, respectively.

**Table 1:** Summary characteristics of the study population

Variables		Study population (%)	Frequency of using rehabilitation services (%)
Sex	Male	493 (64.36)	32(6.62)
	Female	273 (35.64)	28(10.61)
	Total	766	60(7.83)
	Missing value	20	-
Age groups (years)	18–27	69 (10.66)	6(8.95)
	28–37	218 (33.69)	18(8.49)
	38–47	241 (37.25)	17(7.23)
	48–57	98(15.15)	6(6.31)
	> = 58	21 (3.25)	2(9.52)
	Total	647	49(7.57)
	Missing value	124	-
Marital status	Single	403 (54.02)	28(7.11)
	Married	301 (40.35)	22(7.43)
	Widowed and divorced	42 (5.63)	4(10.01)
	Total	746	54(7.23)
	Missing value	40	-
Place of residence	Urban setting	681 (88.33)	58(8.76)
	Rural setting	90 (11.67)	2(2.22)
	Total	771	60(7.78)
	Missing value	15	-
Head of households	No	418 (54.71)	35(8.57)
	Yes	346 (45.29)	24(7.07)
	Total	764	59(7.72)
	Missing value	22	-
Disability severity	1^st^ quartile(low severity)	259(39.72)	22(8.56)
	2^nd^ quintile	125(19.17)	5(4.06)
	3^rd^ quintile	165(25.31)	3(3.12)
	4^th^ quintile(high severity)	103(15.80)	22(13.17)
	Total	652	52(7.97)
	Missing value	134	-
Insurance	No insurance	83 (10.91)	9(11.11)
	Social Security	331 (43.50)	26(8.02)
	Military	21 (2.76)	2(9.52)
	Universal Health Insurance	189 (24.84)	13(7.02)
	Civil Servants	91(11.96)	5(5.68)
	Other	46(6.04)	4(8.69)
	Total	761	59(7.75)
	Missing value	25	-
SES	1^st^ quintile (the lowest)	120 (20.13)	10(8.33)
	2^nd^ quintile	120 (20.13)	6(5.08)
	3^rd^ quintile	118 (19.80)	4(3.38)
	4^th^ quintile	120 (20.13)	16(13.55)
	5^th^ quintile (the highest)	118 (19.80)	20(17.24)
	Total	596	56(9.39)
	Missing value	170	-

In this study, the value of the C was estimated 0.25 (*p*-value = 0.025, Standard error = 0.114) that shows URS was unequally distributed, and concentrated among the higher SES groups.

Table 2 shows the results of decomposition analysis of socioeconomic inequalities in URS in the study population. The SES was the largest contributing factor and explained 94.22% of the overall socioeconomic inequality in URS. Following the SES, age (rural or urban setting) was the second-largest contributor and explained 31.04% of the overall socioeconomic inequality in URS. Also, marital status had a positive influence on the overall inequality, and it explained 11.26% of the observed inequality in URS in PWD.

**Table 2:** Decomposition of socioeconomic inequality in poor access to rehabilitation services in the study population

Variables	Partial effects	Mean	Elasticity	Concentration Index ( C_k_)	Absolute Contribution	Percentage contribution	Summed Percentage Contribution
**Sex**							**-0.03**
Female							
Male	0.004	0.627	0.063	-0.001	0.000	-0.026	
**Age**							**31.04**
18–27							
28–37	-0.027	0.277	-0.187	-0.058	0.011	4.413	
38–47	-0.096	0.306	-0.734	-0.025	0.019	7.471	
48–57	-0.160	0.124	-0.496	-0.051	0.026	10.293	
> = 58	-0.197	0.025	-0.123	-0.177	0.023	8.868	
**Marital status**							**11.26**
Single							
Married	0.138	0.382	1.318	0.021	0.029	11.261	
Others (widow, divorced)	0.000	0.053	0.000	-0.023	0.000	0.000	
**Head of household**							**0.00**
No							
Yes	0.001	0.440	0.011	0.000	0.000	0.000	
**Place of residence**							**0.48**
Rural setting							
Urban setting	0.001	0.874	0.022	0.054	0.001	0.480	
**Disability severity**							**21.25**
1^st^ quartile(low severity)							
2^nd^ quartile	-0.022	0.191	-0.105	-0.050	0.005	2.137	
3^rd^ quartile	-0.106	0.154	-0.408	-0.082	0.035	13.617	
4^th^ quartile (high severity)	0.064	0.256	0.410	0.033	0.014	5.500	
**Insurance coverage**							**-13.48**
No insurance							
Social Security	-0.063	0.434	-0.684	0.012	-0.009	-3.338	
Military	0.041	0.027	0.028	0.386	0.011	4.347	
Universal Health Insurance	-0.040	0.248	-0.248	-0.172	0.044	17.357	
Civil Servants	-0.072	0.119	-0.214	0.354	-0.079	-30.854	
Other	0.014	0.060	0.021	-0.116	-0.003	-0.991	
**SES**							**94.22**
1st quintile ( the lowest)							
2nd quintile	-0.026	0.201	-0.131	-0.391	0.053	20.786	
3rd quintile	-0.072	0.197	-0.355	0.006	-0.002	-0.866	
4th quintile	0.018	0.201	0.090	0.405	0.038	14.906	
5th quintile (the highest)	0.037	0.197	0.182	0.801	0.152	59.392	
**Explained**					**0.37**		**144.75**
**Residuals**					**-0.12**		**-44.75**
**Total**					0.25		**100.00**

## Discussion

In this cross-sectional population-based study, we applied the concentration index to investigate SES gradients for URS in Iran. Our findings indicated that URS was concentrated among the higher SES individuals as a whole. Regarding that long-term rehabilitation services are not covered by health insurance in Iran, people in the lower SES groups face financial problems to use such services. Financial problems is one of the main barriers to access to healthcare among PWD in Iran. The study by Karami Matin et al. indicated that 88% of PWDs experienced financial problems to access healthcare and 41.9% had to borrow money to pay for such healthcare [49].

The results of decomposition analysis indicated that the SES was the largest contributor to the socioeconomic inequality in URS. The finding explains that the SES has a leading role in the unequal distribution of URS among adults with disabilities. Regarding the lack of insurance coverage for some necessary therapies like speech therapy, occupational therapy and orthotics and prosthetics in Iran, socioeconomic status could be an essential factor to utilize such services. For example, Abdi et al. found that lack of affordability was one of the main barriers to utilize rehabilitation services in Iran [12]. Moreover, the findings of Soltani et al. showed that individuals in the low income groups were less likely to access to healthcare among PWD in Iran [2].

In other countries, some studies show that individuals with middle and high socio-economic status are more likely willing to pay for rehabilitation (e.g. physiotherapy) compared with low socio-economic status [50]. There are similar findings about home-based rehabilitation as well. For example, a study by Li et al. indicated that older adults who had a higher income, had at least one partner who worked, and had health insurance are willing to pay more for home-based rehabilitation services [51].

Also, the role of private for profit providers in providing therapies and rehabilitation services is outstanding in Iran that impose high health costs on families. Public financing for rehabilitation services is necessary to make sustainable progress towards universal health coverage. These funds need to be used effectively to assure equitable access to high-quality healthcare and financial protection for PWD. Thus, without financial access to rehabilitation services, PWD may not be able to return to work or participate in other activities of daily living.

In the present study, age was the second major contributor to the socioeconomic inequality in URS. The positive contribution results from both the negative C for age groups and the negative elasticity of all measures of URS. In other words, the participants in lower age groups are more likely to be wealthy and have better access to rehabilitation services than their counterparts in higher age groups. The result implies that young people with disability are more likely to seek and access to rehabilitation services compared to those in higher age groups. A study by Maart and Jelsma indicated that PWD over 65 years old were less likely to have the medical rehabilitation that they required [52]. Also, a study in Brazil indicated that access to rehabilitation was more prevalent in people aged 0 to 17 years among PWD, and those in higher socioeconomic status [53].

In the present study, the severity of disability was the third major contributor to the socioeconomic inequality in URS. The positive contribution is due to both the positive C for the severity of disability and the positive elasticity of all measures of URS. The finding indicates that the participants with more severe disability were more likely to be wealthy and to use rehabilitation services than the others. This result reveals that people with more severe disability probably face more unmet healthcare needs than those with a mild disability. The study by Sakellariou and Rotarou, in the United Kingdom, indicated that individuals with a severe disability were more likely to face a health problem than those with a mild disability [54].

A study by Ahmadzadeh et al. shows that there are significant inequalities in the geographical distribution of outpatient rehabilitation services in Iran [9]. For example, they found that the rate of occupational therapy and physiotherapy offices in Tehran was 35.5 and 104.32 per 1,000,000 population while provinces like Hormozgan and North Khorasan had the lowest rate of occupational offices (0.6 per 1,000,000) and physiotherapy offices (14.8 per 1,000,000) in Iran, respectively. Also, there was a higher proportion of speech therapy offices in Semnan compared to the Qazvin province (31.20 vs 4.24 per 1,000,000 population).

Although, community-based rehabilitation approach is the only main program for providing rehabilitation services in rural areas in Iran, PWD in rural settings still face barriers to access to outpatient rehabilitation services [55–57]. Problems in the referral system especially at the top level, reluctance to apply for the Welfare Organization due to negative attitudes, lack of insurance coverage, poorer-quality services in the public sector, and long waiting times in public rehabilitation centers may be other major reasons for poorer access to rehabilitation services in rural areas [3, 55, 58].

Overall, our findings suggest that URS is concentrated in well-off PWD. Accordingly, it seems that intersectional actions should be taken to improve financial and geographical access to rehabilitation services for PWD. Since rehabilitation services are essential for improving body function and quality of life for PWD, changing landscape of health insurance coverage is recommended to promote equality in URS. In addition, there are other factors such as treatment duration, patient satisfaction, innovation technology, cultural characteristics, availability, etc. that may affect access to rehabilitation services, and should to be addressed in the future.

Coverage for therapy and rehabilitation services have received little attention over the past several decades in Iran. Studies shows that lack of insurance coverage for rehabilitation services cause lack of access or long appointment delay for PWD in Iran. Since, therapies as long term services can impose catastrophic costs on PWD and families, health policy reforms are necessary to ensure the health rights and good health of disadvantaged groups in Iran.

In addition to therapy and rehabilitation services, PWD need other health services such as assistive technologies, medicines, medical visits, etc. that can exacerbate financial problems for PWD. Assistive technologies such as hearing aids, wheelchairs, orthotics and prosthetics are one of the main needs of PWD that have no insurance coverage. Regarding the role and high cost of these technologies, financial protection for PWD should be on the agenda by health policy makers.

## Limitations

The present study had some limitations that should be considered when interpreting the results. In the present study, we used an electronic questionnaire to gather data because of the outbreak of COVID-19 pandemic in Iran. Thus, we think that people without communication tools and those with a low education level (e.g. mobile, computer, etc.) probably have had a lower chance of participation in the study. In the light of these shortcomings, it is suggested to interpret results with caution.

Also, PWD under 18 years old had not participated in the study that may affect the value of socioeconomic inequalities in access to rehabilitation services. In this study, only 3.17% of participants were above 58 years old. Regarding the high prevalence of disabilities among older people [6, 59, 60], we suggest further research on socioeconomic related inequalities in access to rehabilitation services among the elderly population.

In addition, a low percent of the study participants settled in rural area that may have a poorer financial and physical access to rehabilitation compared their counterparts in urban area. Our findings suggest more studies on the availability and quality of rehabilitation services in rural area in Iran. However, Ahmadzadeh et al. reveals that there is a significant difference in the distribution of outpatient rehabilitation facilities in Iran [9].

Another limitation in the present study was related to the cross-sectional nature of the study and the lack of detailed clinical information about the disability status of the study population. Also, self-reporting of disability status was another limitation in the present study. Self-reporting is a common challenge in population studies that may cause response bias in the study. Furthermore, different factors such as geographical features, physical access, cultural factors, and availability of healthcare may contribute to inequality in access to rehabilitation services that can be investigated in future studies.

## Future perspective

Our investigation of inequalities in URS across urban and rural areas has important implications. Improving financial access to rehabilitation services especially for the lower SES groups may reduce socioeconomic-related inequalities in URS in both rural and urban areas. Second, equitable distribution of rehabilitation services need to be on the agenda by key actors to assure access to rehabilitation services for deprived populations. Interventions targeting these populations in terms of improving health outcomes can be a cost-effective strategy to reduce these health disparities.

## Conclusions

Overall, our findings revealed that socioeconomic inequalities in using rehabilitation services was concentrated among well-off PWD. Accordingly, rehabilitation financing through appropriate mechanisms for individuals with low SES is suggested.

## Data Availability

Data and all other materials for this study are kept at the deputy of research and technology of Kermanshah University of Medical Sciences. The datasets generated and/or analyzed during the current study are not publicly available due the terms of consent to which the participants agreed but are available from the corresponding author on reasonable request.

## Abbreviations

URS: Using rehabilitation services
PWD: People with disabilities
C: Concentration Index
CI: Confidence Interval
SES: Socioeconomic Status
PCA: Principal Component Analysis
OOP: Out of pocket payment
ISD: Iranian Society with Disabilities (ISD)
